# Cone-beam computed tomography-guided online adaptive radiotherapy for pharyngeal cancer with whole neck irradiation: dose-volume histogram analysis between adapted and scheduled plans

**DOI:** 10.1093/jrr/rrad103

**Published:** 2024-01-23

**Authors:** Takuya Uehara, Yasumasa Nishimura, Kazuki Ishikawa, Masahiro Inada, Kenji Matsumoto, Hiroshi Doi, Hajime Monzen, Yukinori Matsuo

**Affiliations:** Department of Radiation Oncology, Kindai University Faculty of Medicine, Osaka 589-8511, Japan; Department of Radiation Oncology, Yamatotakada Municipal Hospital, Nara 635-8501, Japan; Department of Radiation Oncology, Kindai University Faculty of Medicine, Osaka 589-8511, Japan; Radiation Therapy Center, Fuchu Hospital, Osaka 594-0076, Japan; Department of Radiation Oncology, Kindai University Faculty of Medicine, Osaka 589-8511, Japan; Department of Radiation Oncology, Nara Prefecture General Medical Center, Nara 630-8581, Japan; Department of Radiation Oncology, Kindai University Faculty of Medicine, Osaka 589-8511, Japan; Department of Medical Physics, Graduate School of Medical Sciences, Kindai University, Osaka 589-8511, Japan; Department of Radiation Oncology, Kindai University Faculty of Medicine, Osaka 589-8511, Japan; Department of Medical Physics, Graduate School of Medical Sciences, Kindai University, Osaka 589-8511, Japan; Department of Radiation Oncology, Kindai University Faculty of Medicine, Osaka 589-8511, Japan

**Keywords:** cone-beam computed tomography-guided online adaptive radiotherapy, adaptive radiotherapy, pharyngeal cancer, intensity modulated radiation therapy, whole neck irradiation, radiotherapy

## Abstract

The present study aimed to evaluate whether an adapted plan with Ethos™ could be used for pharyngeal cancer. Ten patients with pharyngeal cancer who underwent chemoradiotherapy with available daily cone-beam computed tomography (CBCT) data were included. Simulated treatments were generated on the Ethos™ treatment emulator using CBCTs every four to five fractions for two plans: adapted and scheduled. The simulated treatments were divided into three groups: early (first–second week), middle (third–fourth week), and late (fifth–seventh week) periods. Dose-volume histogram parameters were compared for each period between the adapted and scheduled plans in terms of the planning target volume (PTV) (D_98%_, D_95%_, D_50%_ and D_2%_), spinal cord (D_max_ and D_1cc_), brainstem (D_max_) and ipsilateral and contralateral parotid glands (D_median_ and D_mean_). The PTV D_98%,_ D_95%_ and D_2%_ of the adapted plan were significantly higher than those of the scheduled plans in all periods, except for D_98%_ in the late period. The adapted plan significantly reduced the spinal cord D_max_ and D_1cc_ compared with the scheduled plan in all periods. Ipsilateral and contralateral parotid glands D_mean_ of the adapted plan were lower than those of scheduled plan in the late period. In conclusion, the present study revealed that the adapted plans could maintain PTV coverage while reducing the doses to organs at risk in each period compared with scheduled plans.

## INTRODUCTION

The clinical use of intensity modulated radiation therapy (IMRT) represents a significant advancement in radiation oncology. IMRT is effective, especially in patients with head and neck cancer, because the clinical target volume (CTV) generally borders organs at risk (OARs), such as the salivary glands, brainstem and spinal cord. Two randomized controlled trials comparing IMRT and conventional radiation therapy (RT) for patients with early stage nasopharyngeal cancer (NPC) reported significant benefits of IMRT in maintaining salivary function and quality of life of patients [1, 2].

Most investigators use a single IMRT plan for the entire course of treatment as a simultaneous integrated boost (SIB) method [1–5]. However, significant anatomical changes, including shrinkage of the primary tumor or nodal masses and body weight loss, during fractionated RT have been reported in head and neck cancers [6, 7]. Due to potential changes in body contour, target volumes, and OARs during the 7–8 weeks treatment course, the dose distribution to the target volume and OARs may be affected. Therefore, we adopted adaptive two-step IMRT instead of the SIB method for head and neck cancers at our institution [8, 9].

In our adaptive two-step IMRT method, computed tomography (CT) planning was carried out twice: before IMRT for the initial whole neck plan of 46–50 Gy/23–25 fractions, and at the fourth week for the boost IMRT plan of 20–24 Gy/10–12 fractions to the high-risk CTV. However, Bhide *et al*. demonstrated that the most significant volume change and dose alterations occurred at the second week after commencing radiotherapy [10]. Therefore, online adaptive radiotherapy (ART) can overcome the uncertain timing of replanning and variations in tumor and normal tissues.

Ethos™ (Varian Medical Systems, Palo Alto, CA) is a novel commercial linear accelerator that includes an online ART workflow based on high-quality iterative cone-beam CT (iCBCT) images [11]. Ethos™ automatically segments both the target and OARs in daily images and optimizes an entirely new plan to balance tumor coverage with normal tissue sparing. Although several studies have demonstrated that online ART with Ethos™ can lead to clinically acceptable adapted plans for prostate cancer [12, 13], limited previous studies have described the assessment of efficacy in online ART with Ethos™ [14]. Therefore, this study aimed to evaluate whether an online adapted plan with Ethos™ could be used in clinical settings with variable changes in targets and OARs for head and neck cancers, especially pharyngeal cancer.

## MATERIALS AND METHODS

### Patient selection and delivered treatment

This study was approved by our institutional review board (R03–252). Between January and July 2020, 10 patients with NPC, oropharyngeal cancer (OPC), and hypopharyngeal cancer (HPC) who underwent chemoradiotherapy with three cycles of concomitant cisplatin (80.0–100 mg/m^2^ every 3 weeks) at our institution were included in the analysis. The characteristics of the study participants are summarized in Table 1.

**Table 1:** Patient characteristics (*n* = 10)

Case	Age	Gender	Primary site	T stage	N stage	UICC stage (8th)	Total (Boost) RT dose	Concurrent chemotherapy
1	68	Male	NPC	T3	N1	III	70 (24) Gy	Cisplatin
2	62	Male	NPC	T2	N0	II	70 (24) Gy	Cisplatin
3	55	Male	NPC	T1	N3	IVA	70 (24) Gy	Cisplatin
4	54	Female	NPC	T2	N1	II	70 (24) Gy	Cisplatin
5	70	Male	OPC	T3	N1	II	70 (20) Gy	Cisplatin
6	73	Male	OPC	T3	N1	II	70 (20) Gy	Cisplatin
7	69	Male	OPC	T1	N1	I	70 (20) Gy	Cisplatin
8	72	Male	HPC	T2	N0	II	70 (20) Gy	Cisplatin
9	63	Male	HPC	T2	N2b	IVA	70 (20) Gy	Cisplatin
10	63	Male	HPC	T2	N0	II	70 (20) Gy	Cisplatin

UICC = The Union for International Cancer Control, NPC = nasopharyngeal cancer, OPC = oropharyngeal cancer, HPC = hypopharyngeal cancer.

The gross tumor volume (GTV) included any visible disease on imaging, using magnetic resonance imaging, CT or positron-emission tomography/CT and physical examination [8, 9, 15, 16]. The primary CTV encompassed a 5.0–10.0 mm margin with appropriate anatomical correction around the primary GTV. The nodal CTV was defined and delineated in accordance with the Danish Head and Neck Cancer Group, European Organisation for Research and Treatment of Cancer, French Group of Radiation Oncology for Head and Neck Cancer, French Head and Neck Cancer Group, National Cancer Institute of Canada and Radiation Therapy Oncology Group consensus guidelines [17]. Cervical lymph nodes with the shortest axial diameters of 10 mm or more (5 mm for retropharyngeal nodes) were defined as metastatic. The pharyngeal region for each type of pharyngeal cancer, bilateral Level II–IV nodes and the retropharyngeal nodes were included in the CTV for whole-neck irradiation. Level Ib was included in the CTV only when submandibular lymph nodes were involved. Margins of 3.0–4.0 mm for the treatment setup and internal organ motion errors were added to the CTV to determine the planning target volume (PTV). For the planning volume for OAR, a 3.0 mm margin was added to the brainstem and spinal cord [18].

A two-step IMRT method was used for the delivery of radiotherapy to the 10 patients [8, 9]. In the initial plan, 46–50 Gy in 23–25 fractions were delivered to the initial whole-neck PTV. Thereafter, a boost plan was administered to the high-risk CTV, up to a total dose of 70 Gy in 35 fractions. All patients underwent treatment using a Halcyon™ linac (Varian Medical Systems, Palo Alto, CA) with daily iCBCT scans.

### Treatment emulation

In the present study, all reference plan generation in the Ethos™ emulator was created with the initial plans for the whole neck region using 12 equidistant field IMRT. The prescribed dose was 70 Gy and normalized to a dose of 68.6 Gy (98% dose) to 95% of the PTV. Our goals and acceptable criteria for dose-volume histogram (DVH) parameters are shown in Table 2. Simulated treatment was performed on the Ethos™ treatment emulator, a nonclinical version of the Ethos™ software, weekly (once every four to five fractions) for each patient using iCBCTs. The entire dataset consisted of 70 simulated fractions (seven fractions per patient). The dataset was divided into three groups for analysis: early (first–second week, 20 CBCT scans), middle (third–fourth week, 20 CBCT scans), and late (fifth–seventh week, 30 CBCT scans) periods.

**Table 2:** Our DVH goal and acceptable criteria for PTV and OARs

	Parameter	DVH goal	Acceptable criteria
PTV^a^	D_98%_	>93%	>90%
	D_95%_	= 98%	>96%
	D_50%_	<105%	<107%
	D_2%_	<120%	<125%
Spinal cord	D_max_	<50.0 Gy	<54.0 Gy
	D_1cc_	<46.0 Gy	<50.0 Gy
Brainstem	D_max_	<54.0 Gy	<64.0 Gy
Parotid gland^b^	D_median_	<20.0 Gy	<24.0 Gy
	D_mean_	<26.0 Gy	<30.0 Gy

DVH = dose-volume histogram, PTV = planning target volume, OARs = organs at risk, D_*x*%_ = dose to *x*% of the volume, D_max_ = maximum dose, D_1cc_ = dose to 1cc of the volume, D_median_ = median dose, D_mean_ = mean dose.

^a^100% doses were set to 70 Gy.

^b^At least one parotid gland

Additionally, to evaluate ART in Ethos™ with boost settings, another reference plan was created for a boost plan for the high-risk CTV using 12 equidistant field IMRT. To make it the same as the clinical setting at our institution, the prescribed dose was 70 Gy and normalized to a dose of 68.6 Gy (98% dose) to 95% of the boost PTV, and the boost plan should satisfy the same criteria for DVH parameters (Table 2).

### Adaptive workflow

The adaptation process on Ethos™ emulator includes auto-segmentation of the daily anatomy, calculation of the dose in the scheduled plan (dose from the non-adapted plan on the daily anatomy), and optimization and calculation of the dose in the adapted plan. The scheduled plan is the reference plan, but it is a recalculation of the auto-segmentation of the daily anatomy using CBCT. All calculations were performed using Acuros XB. Both the scheduled and adapted plans used the field geometry of the reference plan. Particularly with the adapted plan, clinical goals of the adapted plan for optimization were the same as those of the reference plan. The auto-segmentation, adapted dose and scheduled dose for each fraction were exported to our clinical treatment planning system (TPS), Eclipse (Varian Medical Systems, Palo Alto, USA), and the DVH parameters were assessed. In terms of the auto-segmentation, the consistency of each volume was verified by two expert radiation oncologists for all fractions.

### Analysis of DVH parameters

The comparison of DVH parameters between the adapted and scheduled plans focused on D_98%_, D_95%_, D_50%_ and D_2%_ for the PTV, where D_98%_, D_95%_, D_50%_ and D_2%_ were the doses received by 98, 95, 50% and 2% of the PTV, respectively. In the present study, the DVH parameters of the spinal cord, brainstem and parotid glands were evaluated because these three OARs were the most important for head and neck IMRT plans: D_max_ (maximum dose) and D_1cc_ (doses received by 1 cc of the spinal cord) of the spinal cord; D_max_ of the brainstem; and D_median_ (median dose) and D_mean_ (mean dose) of the ipsilateral and contralateral parotid glands, respectively. Paired *t*-tests were used to identify differences between the adapted and scheduled plans. The DVH parameters of the adapted and scheduled plans for the initial whole-neck plan were compared to those in the early, middle and late periods. In addition, the DVH parameters of the adapted and scheduled plans for boost setting were evaluated in the late period. All analyses were performed using JMP software (version 14.0.0; SAS Institute, Cary, NC), and differences were considered statistically significant at *P* < 0.05.

## RESULTS

The DVH parameters of the reference plans for each type of pharyngeal cancer are summarized in Table 3. All these parameters met our goal and acceptable criteria (Table 2).

**Table 3:** DVH parameters of reference plan for each type of pharyngeal cancer

	Parameter	Primary site		
		NPC	OPC	HPC
PTV	D_98%_	64.9 ± 1.9	66.5 ± 0.4	67.2 ± 0.6
	D_95%_	68.5 ± 0.3	68.7 ± 0.1	68.7 ± 0.1
	D_50%_	70.9 ± 0.8	70.9 ± 0.4	70.6 ± 0.2
	D_2%_	72.8 ± 1.5	72.8 ± 0.8	72.1 ± 0.2
Spinal cord	D_max_	44.1 ± 1.1	43.2 ± 0.5	43.3 ± 3.0
	D_1cc_	40.4 ± 1.0	38.9 ± 0.5	39.5 ± 1.9
Brainstem	D_max_	43.6 ± 4.7	41.1 ± 1.1	36.4 ± 1.9
Ipsilateral parotid gland	D_median_	20.6 ± 4.9	21.5 ± 4.4	18.2 ± 1.7
	D_mean_	27.8 ± 5.7	31.2 ± 5.1	26.1 ± 0.9
Contralateral parotid gland	D_median_	16.4 ± 2.9	14.7 ± 1.4	12.8 ± 2.0
	D_mean_	22.0 ± 4.5	21.3 ± 2.3	20.7 ± 1.5

Abbreviations are the same as indicated in Tables 1 and 2.

Auto-segmentation of all fractions was verified by two expert radiation oncologists, and no editing was deemed necessary for all cases. The quality of Ethos™ auto-segmentation was satisfactory for clinical use.

The volumes of GTV, CTV and PTV for planning CT and each period were evaluated and summarized in Table 4. The volume of GTV, CTV and PTV tends to decrease gradually over time, especially OPC.

**Table 4:** The volume of GTV, CTV and PTV for each type of pharyngeal cancer

	Primary site	At planning CT	Early period (1st-2nd week)	Middle period (3rd-4th week)	Late period (5th–7th week)
		Volume	Volume	Volume	Volume
GTV	NPC	94 ± 68	96 ± 68	94 ± 62	92 ± 57
	OPC	67 ± 44	68 ± 48	65 ± 46	65 ± 41
	HPC	29 ± 19	29 ± 16	29 ± 16	29 ± 16
CTV	NPC	422 ± 202	416 ± 191	407 ± 173	404 ± 156
	OPC	431 ± 112	438 ± 92	418 ± 89	415 ± 78
	HPC	291 ± 75	293 ± 72	293 ± 64	291 ± 66
PTV	NPC	701 ± 266	687 ± 250	674 ± 230	669 ± 208
	OPC	680 ± 129	675 ± 104	651 ± 105	648 ± 95
	HPC	503 ± 96	501 ± 89	499 ± 82	497 ± 84

UICC = The Union for International Cancer Control, NPC = nasopharyngeal cancer, OPC = oropharyngeal cancer, HPC = hypopharyngeal cancer.

The DVH parameters of the adapted and scheduled plans for each period are summarized in Table 5. PTV D_98%_ of the adapted plan in the early and middle periods were 66.0 and 65.8 Gy, respectively. These values were significantly higher than those of the scheduled plan (64.3 and 65.3 Gy; *P* = 0.02 and < 0.01, respectively). The PTV D_95%_ of the adapted plan in the early, middle and late periods were 68.7, 68.7 and 68.5 Gy, respectively. These values were significantly higher than those of the scheduled plan (67.6, 68.1 and 68.1 Gy; *P* < 0.01 for all, respectively). The PTV D_2%_ values of the adapted plan in the early, middle and late periods were 72.6, 72.6 and 72.8 Gy, respectively. These values were significantly lower than those of the scheduled plan (73.0, 72.9 and 73.2 Gy; *P* = 0.04, 0.04 and 0.02, respectively). No significant difference in the D_50%_ of the PTV was observed between the adapted and scheduled plans in all periods.

**Table 5:** DVH parameters for adapted and scheduled plan in each period

	Parameter	Early period (1st–2nd week)		*P*-value	Middle period (3rd–4th week)		*P*-value	Late period (5th–7th week)		*P*-value
		Adapted plan	Scheduled plan		Adapted plan	Scheduled plan		Adapted plan	Scheduled plan	
PTV	D_98%_	66.0 ± 1.8	64.3 ± 3.4	0.02	65.8 ± 1.9	65.3 ± 1.5	<0.01	64.3 ± 5.2	64.8 ± 2.2	0.54
	D_95%_	68.7 ± 0.1	67.6 ± 1.5	<0.01	68.7 ± 0.1	68.1 ± 0.5	<0.01	68.5 ± 0.6	68.1 ± 0.6	<0.01
	D_50%_	70.7 ± 0.5	70.7 ± 0.7	0.76	70.7 ± 0.5	70.9 ± 0.6	0.08	70.8 ± 0.6	71.0 ± 0.6	0.14
	D_2%_	72.6 ± 0.9	73.0 ± 1.1	0.04	72.6 ± 1.0	72.9 ± 0.9	0.04	72.8 ± 1.4	73.2 ± 1.1	0.02
Spinal cord	D_max_	42.6 ± 1.7	45.9 ± 3.5	<0.01	43.0 ± 1.7	46.9 ± 5.0	<0.01	43.2 ± 2.0	48.3 ± 6.0	<0.01
	D_1cc_	38.9 ± 1.1	40.2 ± 1.8	<0.01	39.1 ± 1.7	41.4 ± 3.2	<0.01	39.4 ± 1.7	42.4 ± 4.0	<0.01
Brainstem	D_max_	40.1 ± 3.3	40.9 ± 5.7	0.47	39.9 ± 4.0	40.8 ± 7.0	0.43	40.8 ± 5.3	41.3 ± 5.9	0.47
Ipsilateral parotid gland	D_median_	20.1 ± 3.9	20.3 ± 4.1	0.76	20.6 ± 3.6	20.7 ± 5.1	0.88	21.5 ± 3.9	21.9 ± 6.6	0.12
	D_mean_	28.1 ± 4.8	27.9 ± 4.6	0.71	28.4 ± 4.6	28.7 ± 5.2	0.21	29.0 ± 4.9	30.6 ± 5.9	<0.01
Contralateral parotid gland	D_median_	15.0 ± 3.0	15.0 ± 2.1	0.95	14.9 ± 2.6	14.7 ± 2.5	0.71	15.0 ± 2.8	15.2 ± 2.8	0.52
	D_mean_	21.9 ± 3.5	21.5 ± 2.3	0.29	21.1 ± 3.8	21.4 ± 2.7	0.5	21.8 ± 3.2	22.5 ± 3.3	0.03

DVH = dose-volume histogram, PTV = planning target volume, OARs = organs at risk, D_*x*%_ = dose to *x*% of the volume, D_max_ = maximum dose, D_1cc_ = dose to 1cc of the volume, D_median_ = median dose, D_mean_ = mean dose.

**Fig. 1:**
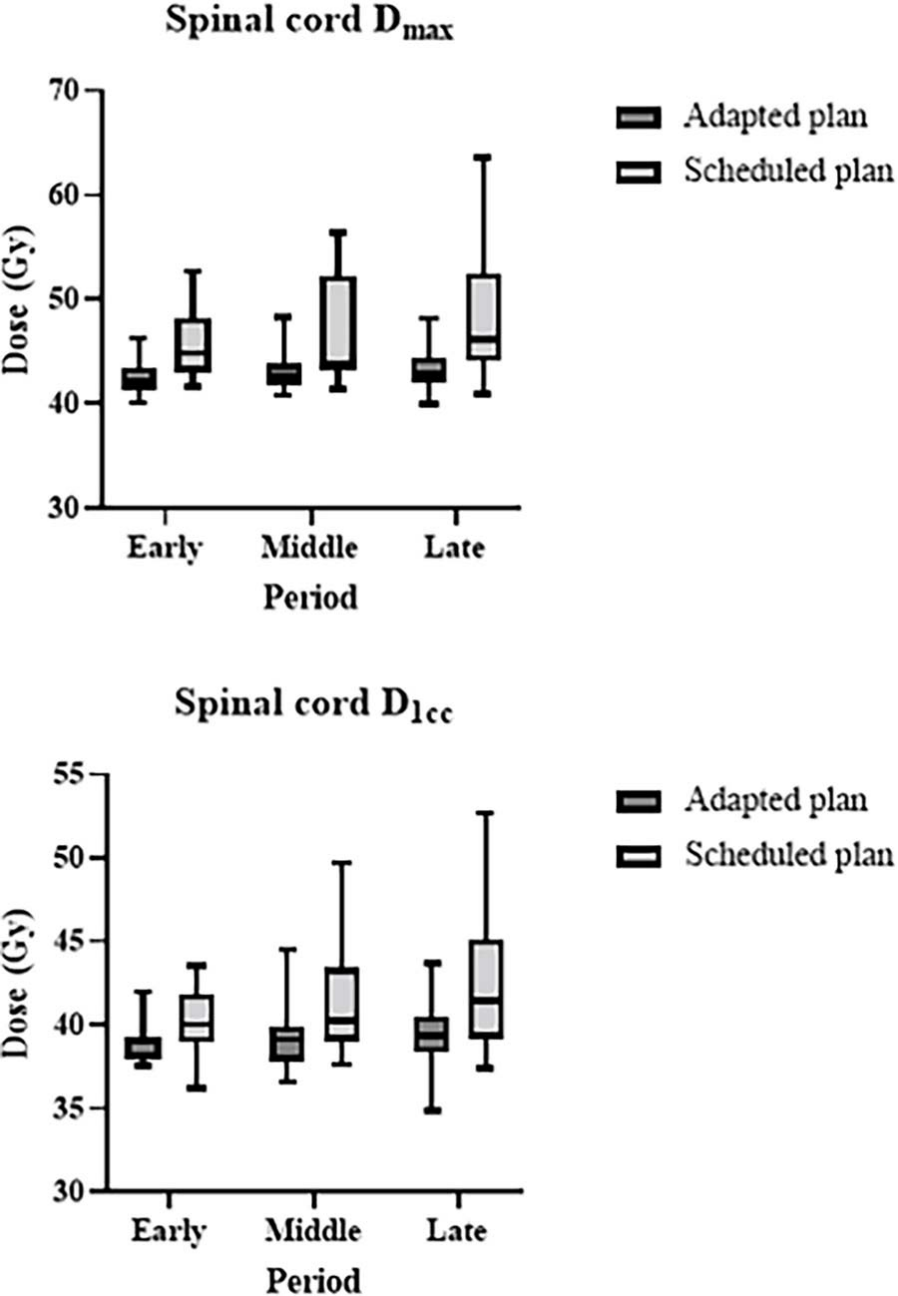
The dosimetric changes of spinal cord D_max_ and D_1cc_ with adapted and scheduled plans were plotted in early, middle and late periods. The central line marks the median, the edges of the box are the 25th and 75th percentiles and the whiskers extend to the adjacent values, which are the most extreme data values that are not outliers.

In terms of the OARs, the spinal cord D_max_ values of the adapted plan in the early, middle and late periods were 42.6, 43.0, and 43.2 Gy, respectively. These values were significantly lower than those of the scheduled plan (45.9, 46.9 and 48.3 Gy; *P* < 0.01 for all, respectively). Similarly, spinal cord D_1cc_ values of the adapted plan were significantly lower than those of the scheduled plan across all periods. The spinal cord D_max_ and D_1cc_ values increased over time, particularly in the scheduled plan (Fig. 1). Regarding the parotid gland, the ipsilateral and contralateral parotid glands D_mean_ of the adapted plan (29.0 and 21.8 Gy) were significantly lower than those of the scheduled plan (30.6 and 22.5 Gy) in late period, respectively (*P* < 0.01 and 0.03, respectively).

**Fig. 2:**
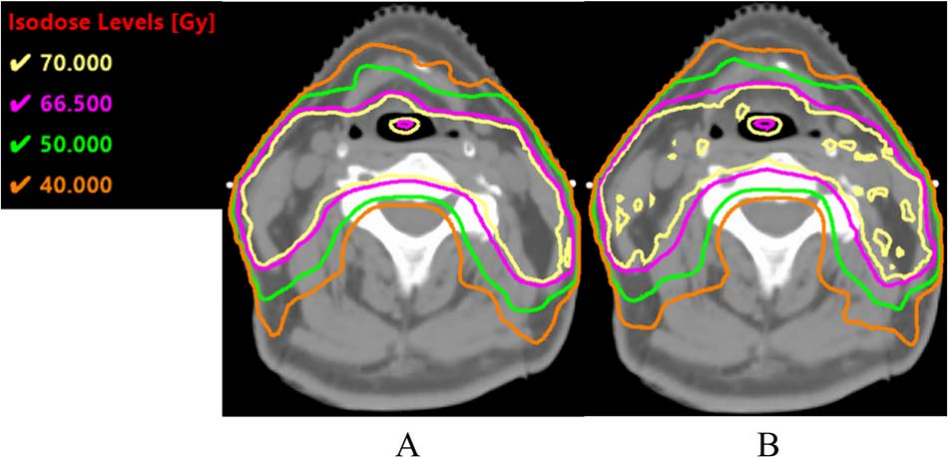
Dose distributions in the sixth week of a patient with right tonsil cancer for the scheduled plan (**A**) and adapted plan (**B**). Yellow line, 70 Gy iso-dose line; magenta line, 66.5 Gy iso-dose line; green line, 50 Gy iso-dose line; orange line, 40 Gy iso-dose line.

**Fig. 3:**
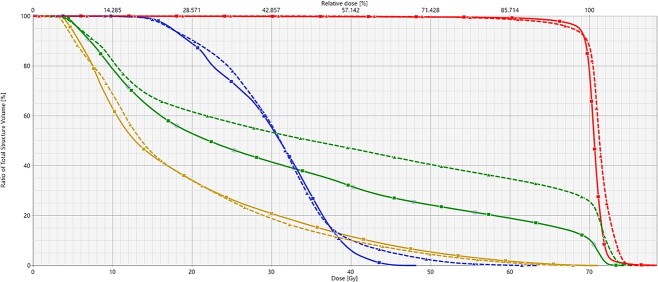
DVHs of the PTV (red), spinal cord (blue), ipsilateral parotid gland (green) and contralateral parotid gland (yellow) for adapted plan (solid lines with squares) and scheduled plan (dashed lines with triangles).

Figures 2 and 3 show the dose distributions and DVH in the sixth week of a patient with right tonsil cancer. In the scheduled plan (Fig. 2A), the spinal cord was included in the high-dose region. In terms of the adapted plan (Fig. 2B), the spinal cord was better spared, while PTV coverage was maintained. In this particular patient, the spinal cord D_max_ and D_1cc_ values for the scheduled plan were 63.6 Gy and 52.7 Gy, respectively. These D_max_ and D_1cc_ values did not meet our acceptable criteria (Table 2); thus, the scheduled plan was clinically unacceptable. In contrast, the spinal cord D_max_ and D_1cc_ values for the adapted plan were 48.2 and 43.6 Gy, respectively. In addition, the ipsilateral parotid glands were better spared by the adapted plan than by the scheduled plan, and no difference was observed for the contralateral parotid glands. Thus, for this patient, the adapted plan was clinically acceptable.

In addition, the DVH parameters for the adapted and scheduled plan with boost settings in the late period (fifth–seventh week) are summarized in Table 6. Boost PTV D_98%_ and D_95%_ of the adapted plan were 66.9 and 68.7 Gy, respectively. These values were significantly higher than those of the scheduled plan (65.2 and 67.9 Gy; *P* < 0.01, respectively). No significant difference in the D_50%_ and D_2%_ of the boost PTV was observed between the adapted and scheduled plans. On the other hand, the ipsilateral parotid glands D_median_ and D_mean_ of the adapted plan (12.3 and 18.6 Gy) were significantly lower than those of the scheduled plan (13.5 and 20.1 Gy), respectively (*P* < 0.01). No significant difference in the spinal cord, brainstem and contralateral parotid gland was observed between the adapted and scheduled plans.

**Table 6:** DVH parameters for adapted and scheduled plan with boost setting in late period (5th–7th week)

	Parameter	Late period (5th–7th week)		*P*-value
		Adapted plan	Scheduled plan	
PTV	D_98%_	66.9 ± 1.6	65.2 ± 2.6	<0.01
	D_95%_	68.7 ± 0.1	67.9 ± 1.0	<0.01
	D_50%_	70.3 ± 0.4	70.4 ± 0.5	0.43
	D_2%_	71.8 ± 0.8	72.0 ± 2.0	0.65
Spinal cord	D_max_	40.3 ± 4.0	42.1 ± 6.5	0.06
	D_1cc_	34.7 ± 5.3	34.6 ± 6.3	0.93
Brainstem	D_max_	28.7 ± 18.9	29.3 ± 19.4	0.08
Ipsilateral parotid gland	D_median_	12.3 ± 7.1	13.5 ± 8.4	<0.01
	D_mean_	18.6 ± 9.1	20.1 ± 10.0	<0.01
Contralateral parotid gland	D_median_	10.7 ± 6.8	10.8 ± 6.5	0.81
	D_mean_	12.9 ± 7.6	13.2 ± 7.5	0.24

DVH = dose-volume histogram, PTV = planning target volume, OARs = organs at risk, D_*x*%_ = dose to *x*% of the volume, D_max_ = maximum dose, D_1cc_ = dose to 1cc of the volume, D_median_ = median dose, D_mean_ = mean dose.

## DISCUSSION

In this study, adapted and scheduled plans for pharyngeal cancer with an initial whole-neck plan using the Ethos™ emulator were evaluated. Our study showed that the adapted plans could maintain PTV coverage while reducing the dose to OARs in each period compared to scheduled plans. To the best of our knowledge, this is the first study to demonstrate that weekly online adapted plans can create dosimetrically improved plans for pharyngeal cancer with whole-neck irradiation concerning PTV and OARs.

Regarding the PTV, D_98%_, D_95%_ and D_2%_ of the adapted plan were better than those of the scheduled plan in almost all periods. Boost PTV D_98%_ and D_95%_ of the adapted plan were better than those of the scheduled plan. One reason for this might be that the priority of PTV coverage was higher in the clinical goal and was optimized every time for the adapted plan. Especially in PTV D_95%_ of the adapted plan, these values were maintained near 68.6 Gy every time with appropriate normalization, normalized to the dose of 68.6 Gy (98% dose) to 95% of the PTV in this study. Additionally, the volumes of GTV, CTV and PTV for planning CT and each period are summarized in Table 4. The volume of GTV, CTV and PTV may have decreased over time, especially OPC. Another reason for better PTV coverage of the adapted plan might be that the adaptation of anatomical changes was properly performed every time for the adapted plan.

In terms of OARs, the spinal cord D_max_ and D_1cc_, as well as the ipsilateral and contralateral parotid gland D_mean_ of the adapted plan, were significantly lower than those of the scheduled plan. In the boost setting, the ipsilateral parotid glands D_median_ and D_mean_ of the adapted plan were significantly lower than those of the scheduled plan. Regarding dosimetric changes according to changes in body surface contour and positional changes in target and risk organs for head and neck cancer, significant changes in the maximum dose to the spinal cord and the dose to 50% of the volume of the parotid glands were reported by Ahn *et al*. [19]. They concluded that adaptive re-planning of head and neck IMRT is necessary. In the present study, the spinal cord D_max_ and D_1cc_ and ipsilateral parotid gland D_mean_ values increased over time (Table 5, Fig. 1). Nishi *et al*. described that the mean dose to the parotid glands and dose to 2% of the volume of the spinal cord increased significantly in boost IMRT plans with CT of the third or fourth week [7]. As a two-step IMRT method that can adjust to anatomical changes in the body surface contour and target and risk organs during IMRT treatment, this method was effective in preventing any increase in the high-dose regions of the spinal cord and parotid glands. However, Bhide *et al*. demonstrated that the most significant volume changes and dose alteration occur in the second week after commencing radiotherapy [10]. Therefore, weekly online ART may be necessary for tumor and normal tissue variations with pharyngeal cancer. In addition, dose–volume relationships of the spinal cord with the incidence of Lhermitte’s sign after radiotherapy have been described in previous studies [20, 21]. In this study, the adapted plans reduced spinal cord D_max_ and D_1cc_ compared with the scheduled plans. Nishimura *et al*. demonstrated that the incidence of Grade 1 myelitis was 10% [9]. A reduction in the spinal cord dose may contribute to reduced toxicity.

In this study, all reference plan generation in the Ethos™ emulator was created with the initial plans for the whole neck region using 12 equidistant field IMRT. Ethos™ can also create and deliver multi-arc volumetric modulated arc therapy (VMAT) plans, which are commonly used for head and neck cancer. However, VMAT plans take longer to optimize for each adapted fraction, which adds unnecessary treatment delays. In addition, Sibolt *et al*. found that Ethos™ generated IMRT plans resulted in superior dose metrics compared to both 2- and 3-arc VMAT plans generated by Ethos™ [22]. Therefore, 12 equidistant-field IMRT plans were used in this study.

The present study has several limitations, including its retrospective design, relatively small sample size and simulation study. The data in this study are dosimetric data, and therefore further exploration, including clinical outcomes and toxicity, is needed for clinical application and comprehension of online ART with Ethos™ for patients with head and neck cancer. Additionally, the consistency of Ethos™ auto-segmentation accuracy has not been fully reported in previous studies for head and neck cancers. Moazzezi *et al*. [12] and Chapman *et al*. [23] reported that the auto-segmentation results from Ethos™ were accurate enough for prostate cancer. In terms of head and neck cancer, Yoon *et al*. showed that auto-segmentation by Ethos™ was satisfactory [14]. Although in this study, each volume created with Ethos™ auto-segmentation was verified for consistency by two expert radiation oncologists for all fractions, further investigation was needed. However, we believe that the present study provides valuable data that can be reliably adopted in daily clinical practice and serve as the foundation for future clinical trials.

## CONCLUSION

In conclusion, the present study revealed that an adapted plan with Ethos™ can lead to dosimetrically improved plans for pharyngeal cancer with whole neck irradiation. Online ART may be necessary to maintain PTV coverage while reducing OAR doses in all periods of pharyngeal cancer treatment. Weekly online ART could be necessary for variations in tumor and normal tissues with pharyngeal cancer starting approximately the second week.
